# Lipid Fingerprinting in Mild versus Severe Forms of Gestational Diabetes *Mellitus*

**DOI:** 10.1371/journal.pone.0144027

**Published:** 2015-12-03

**Authors:** Bárbara Yasmin Gueuvoghlanian-Silva, Fernanda Bertuccez Cordeiro, Thalita Frutuoso Lobo, Thaís Regiani Cataldi, Edson Guimarães Lo Turco, Ricardo Pimenta Bertolla, Rosiane Mattar, Maria Regina Torloni, Silvia Daher

**Affiliations:** 1 Department of Obstetrics, Universidade Federal de São Paulo, Sao Paulo, SP, Brazil; 2 Human Reproduction Section, Division of Urology, Department of Surgery, Universidade Federal de São Paulo, Sao Paulo, SP, Brazil; 3 Department of Genetics ESALQ-USP, University of Sao Paulo, Piracicaba, SP, Brazil; East Tennessee State University, UNITED STATES

## Abstract

The blood serum lipid profile of women with Gestational Diabetes *Mellitus* (GDM) is still under study. There are no data on the serum lipid profile of GDM patients with more severe (insulin treated) compared to milder forms (diet treated) GDM. The aim of our study was to analyze the blood serum lipid profile of patients with milder versus more severe forms of GDM and to compare these findings with those of healthy pregnant women. This cross-sectional analytical study included 30 insulin-treated GDM, 30 diet-only GDM and 30 healthy pregnant women. Serum lipid was extracted from the 90 participants and their lipid profiles were analyzed by lipid fingerprinting using liquid-chromatography-mass spectrometry. A total of 143 parent ions were differentially represented in each of the three groups, belonging to the following classes: Glycerophospholipids, Sterol Lipids, Sphingolipids, Prenol Lipids, Fatty Acyls and Glycerolipids. There were significant differences in the lipid profiles of healthy pregnant women compared to GDM patients and also between milder versus more severe forms of GDM. There are marked differences in lipid fingerprinting between healthy pregnant women compared to those with GDM in the third trimester. Moreover, the lipid profile of women with more severe forms of GDM differs considerably from that of women with milder forms of GDM. These findings may be useful to help clarify the pathogenesis of milder and more severe forms of GDM.

## Introduction

Gestational Diabetes *Mellitus* (GDM) is the most frequent metabolic disorder of pregnancy, affecting between 1–14% of all women [1]. The prevalence of GDM is expected to increase substantially over the next years with the adoption of new diagnostic criteria recommended by the International Association of Diabetes and Pregnancy Study Groups (IADPSG) and also due to the increasing prevalence of obesity among reproductive age women [2, 3].

The complications associated with GDM can be reduced with adequate glycemic control [4]. While most women with GDM will achieve adequate glycemic control with diet and exercise, a proportion of them will require antenatal insulin treatment (AIT) [5]. The need for insulin characterizes patients with a more severe form of GDM, who will have an increased probability of developing type 2 Diabetes *Mellitus* (T2DM) and cardiovascular complications in the future [6, 7]. These two forms of GDM seem to reflect different degrees of beta cell dysfunction or different pathophysiological mechanisms [8].

During pregnancy, insulin resistance (IR) and hyperlipidemia are important physiological processes that are essential to ensure adequate fetal nutrition. In the third trimester of pregnancy, healthy women have major changes in their lipid metabolism that lead to increased plasma triacylglycerol levels and, to a lesser extent, to higher phospholipid and cholesterol levels [9].

Besides acting as a form of energy storage, lipids are an important component of membranes and have many other key functions including their role in signaling pathways and the regulation of other molecules. Imbalances in lipid signaling pathways are associated with inflammation progression, autoimmunity and several systemic diseases, such as the metabolic syndrome, atherosclerosis and hypertension [10].

High triglycerides plasma levels are associated with IR/T2DM but this increase is influenced by glycemic levels. Patients with well-controlled T2DM have triglyceride levels similar to healthy controls [11]. In contrast, patients with T2DM, even with adequate metabolic control, have lower HDL cholesterol, free cholesterol levels and phospholipids on the HDL surface [12].

There are conflicting results regarding hyperlipidemia in GDM. While some investigators report significantly higher lipid levels in all trimesters in GDM patients compared to healthy pregnant women, others refute these findings [9, 13]. Herrera and Ortega-Senovilla (2010) highlighted these controversies in their review of studies on the lipid profile of GDM patients [9].

With the advent of new methods, it is now possible to perform more sensitive and specific lipid analyses in extracts of cells and tissues. Lipid fingerprinting is useful because it allows the identification of a lipid profile that could be associated with a specific disease. Liquid chromatography (LC) coupled with electrospray ionization (ESI)-quadrapole time of flight hybrid mass spectrometer (QTOF-MS) allows the identification of complex molecular species [14]. It is well documented that ESI/MS is very useful and efficient for the study of lipids in many diseases. Moreover, this technique allows a direct analysis of the lipid profile of chloroform extracts [15]. Previous investigations in T2DM suggest that this technique is able to detect dyslipidemia associated with that condition [16, 17].

There is evidence indicating that glycemic control affects lipid concentrations. Giuffrida et al. (2012) reported an association between HbA1_c_ levels and changes in lipid profile in patients with type 1 Diabetes *Mellitus* (T1DM) [18]. In contrast, Karkkainen et al. (2013) did not detect significant differences in cholesterol, LDL, HDL and triglyceride levels in samples from third trimester patients with GDM treated with diet or insulin when compared with healthy pregnant women. However, there were significant differences between the groups after delivery [19].

The search for markers that could predict which women will develop the more severe form of GDM has been the focus of several studies [20–22]. This quest is based on the premise that it would be useful to predict which women will develop more severe forms of the disease in order to improve efficiency of health care delivery in GDM and thus optimize maternal and perinatal outcomes in these cases. However, until now, there are no tools that can help to predict this risk and this may in part be due to the lack of studies on possible differences in the physiopathology of milder versus more severe forms of GDM. The aim of our study was to use a lipid fingerprinting approach to analyze the blood serum lipid profile of patients with GDM treated with diet or with insulin, in comparison with healthy pregnant women. To the best of our knowledge, up to the moment, this approach has not been used for lipid analyses in patients with GDM. We hope that our findings will contribute to the understanding of GDM physiopathology and to the future development of new tools for the early prediction of which women will develop more severe forms of the disease.

## Materials and Methods

This cross-sectional analytical study recruited women in the antenatal clinics of two public tertiary teaching facilities in Sao Paulo, Brazil (Dr. Mario de Moraes Altenfelder Silva maternity and Sao Paulo hospital). The study was approved by Ethics in Research Committee of Universidade Federal de São Paulo/Hospital São Paulo (#0704/11) and Ethics in Research Committee of Dr. Mario de Moraes Altenfelder Silva Maternity (#02/12) and written informed consent was obtained from all participants.

The inclusion criteria were singleton pregnancy with a live fetus and gestational age between 30 to 36 6/7 weeks, based on obstetric sonogram performed before the 20^th^ week of pregnancy. Women with any of the following were excluded: pre-existing Diabetes *Mellitus* (type 1 or type 2); chronic systemic autoimmune pre-existing diseases; acute or chronic active infections; solid organ transplant recipients and women using steroids, antibiotics, immunosuppressants, antihistamines or anti-inflammatory medication. Women with obstetric disorders (including but not limited to pre-eclampsia, premature rupture of membranes, preterm labor or placenta previa) were also excluded.

Participants were divided into three groups: healthy pregnant women (controls, n = 30), women diagnosed with GDM adequately controlled with diet (diet-treated GDM, n = 30) and women with GDM that required antenatal insulin treatment (AIT) to control glycemic levels (AIT-GDM, n = 30). All participants had been tested for GDM at 24–28 weeks through a 2-hours, 75g oral glucose tolerance test, as part of their routine antenatal care in the two settings. Women were diagnosed with GDM according to the IADPSG recommendations, i.e. fasting glucose between 92 and < 126 mg/dL and/or 1-hr post 75 g load ≥ 180 mg/dL and/or 2-hr post 75 g load between 153–199 mg/dL [2]. Pre-pregnancy body mass index (BMI) was calculated based on measured height and self-reported pre-pregnancy weight. Race was self-reported.

Upon enrolment, 8 mL of peripheral blood was collected from each participant, regardless of diet, in tubes containing spray-coated silica and a polymer gel (SST) for serum separation (BD Diagnostics). After clot retraction, the sample was centrifuged at 2,205 xg for 10 minutes at room temperature to obtain serum sample, which was immediately frozen at -80°C.

### Lipid extraction

Lipid extraction was performed according to the Bligh and Dyer protocol [23] with some modifications. Briefly, 50 μL of distilled water (HPLC grade- Sigma-Aldrich, St. Louis, MO, USA) was added to 50 μL of serum. Then, 127 μL of chloroform (HPLC grade- Merck, Darmstadt, Germany) and 252 μL of methanol (HPLC grade- Sigma-Aldrich) were added. After homogenization by vortexing, 100 μL of water and 127 μl of chloroform were added for polar and apolar phases separation. The mixture was centrifuged at 800 xg for 5 minutes at room temperature and 200 μL of the lower layer containing lipids were transferred to another LC-MS glass tube (Waters, Milford, MA, USA). After drying the entire liquid content, the samples were stored at -80°C until analysis.

### ESI-(+)-MS analyses

Each sample was analyzed in triplicate. For analysis, the samples were suspended in 100 μL of a 1:1 methanol:water solution and then filtered using a 0.22 μm filter (Merck Millipore Corporation, Billerica, MA, USA). Analyses were performed on a hybrid high-resolution mass spectrometer Q-TOF Ultima Waters (Manchester, England) with Electrospray as the ionization source, in positive mode. The analysis conditions were: source temperature of 150°C, desolvation temperature of 450°C, capillary voltage of 3 kV and cone voltage equal to 35 V. The nitrogen flow in the cone was 50 L.h^-1^ and of desolvation was 550 L.h^-1^. The m/z range acquired was between 500–1200. The equipment was calibrated with a 5 mM sodium formate solution. Chromatography was performed with an Acquity chromatograph (Waters, Manchester, England) with a reverse phase column (Acquity HSS UPLC 1.8 μm, 2.1 x 100 mm) conditioned to 35°C. The flow rate was 0.5 mL.min^-1^. The elution buffers were A: acetonitrile doped with 0.1% formic acid and B: water with 0.1% formic acid.

### Data analysis

MassLynx 4.1 software (Waters, Manchester, UK) was used for the preprocessing of mass spectra obtained by ESI-MS. Each sample spectrum was processed for background removal and smoothing and for peaks centroiding.

The intensity values were normalized by cubic Pareto scaling and scaling transformation. Statistical analysis was performed using MetaboAnalyst 2.0 software (http://www.metaboanalyst.ca). Univariate (One-way ANOVA) and multivariate (principal component analysis [PCA] and discriminant analysis by partial least squares [PLS-DA]) analyses workflows were applied to the data set. An alpha error of 5% was adopted.

The PLS-DA models were built and the variable importance in the projection (VIP) was used to identify the ions that had greater discriminatory effect between the groups in the component with higher power projection. Considering the masses of the differentially expressed ions, the lipids were identified with the aid of SimLipid 3.0 software (PREMIER Biosoft, Palo Alto, CA, USA), with a maximum acceptable error of 50ppm, positive polarity (M+H, M+Na and M+K) and selecting only the following categories: Glycerophospholipids (GP), Sphingolipids (SP), Fatty Acyls (FA), Glycerolipids (GL), Sterol Lipids (ST) and Prenol Lipids (PR). Moreover, the criteria for the selection of the best lipid category of each ion were: between H^+^ and Na^+^ polarity, we chose the one with the lowest mass error; the K^+^ polarity was chosen only in cases where there were no H^+^ or Na^+^ polarity ions, and in these cases, the one with the lowest mass error.

The chi-square test was used to compare categorical variables. The Kolmogorov–Smirnov or Shapiro–Wilk tests and Skewness and Kurtosis values were used to assess the distribution of continuous numerical variables. For analysis of variance between groups, the one-way ANOVA test was used, followed by a Tukey post-hoc test. All tests were considered significant at p<0.05. Statistical analyses were performed with standard software (GraphPad Prism 5 for Windows).

## Results

Table 1 presents the main characteristics of the 90 participants. Healthy controls were significantly younger than diet-treated GDM patients (p<0.01) and patients with AIT-GDM (p<0.0001). Controls were also significantly leaner than GDM patients (p<0.0001).

**Table 1:** Main characteristics of 90 pregnant women.

Variable	Controls N = 30	Diet-treated GDM N = 30	Insulin-treated GDM N = 30	p
Race				
White	10 (33%)	13 (43%)	12 (40%)	
Mixed	16 (53%)	14 (47%)	12 (40%)	0.73^*a*^
Black	4 (14%)	3 (10%)	6 (20%)	
Age (years)				
Mean	27.2	32.9^*c*^	33.0^*d*^	<0.0001^*b*^
SD	5.2	6.1	5.2	
Pre-pregnancy BMI (Kg/m^2^)				
Mean	23.9	28.2^*d*^	30.9^*d*^	<0.0001^*b*^
SD	5.6	4.9	5.7	
Parity				
nulliparous	9 (30%)	9 (30%)	6 (20%)	0.60^*a*^
multiparous	21 (70%)	21 (70%)	24 (80%)	
GA at collection (weeks)				
Mean	32.9	33.2	32.9	0.76^*b*^
SD	1.9	1.9	1.9	

GDM: Gestational Diabetes *Mellitus*, SD: Standard deviation, BMI: Body Mass Index, GA: Gestational age

^*a*^Chi-square test

^*b*^One-way ANOVA test

^*c*^p<0.01 versus Control

^*d*^p<0.0001 versus Control

**Fig 1:**
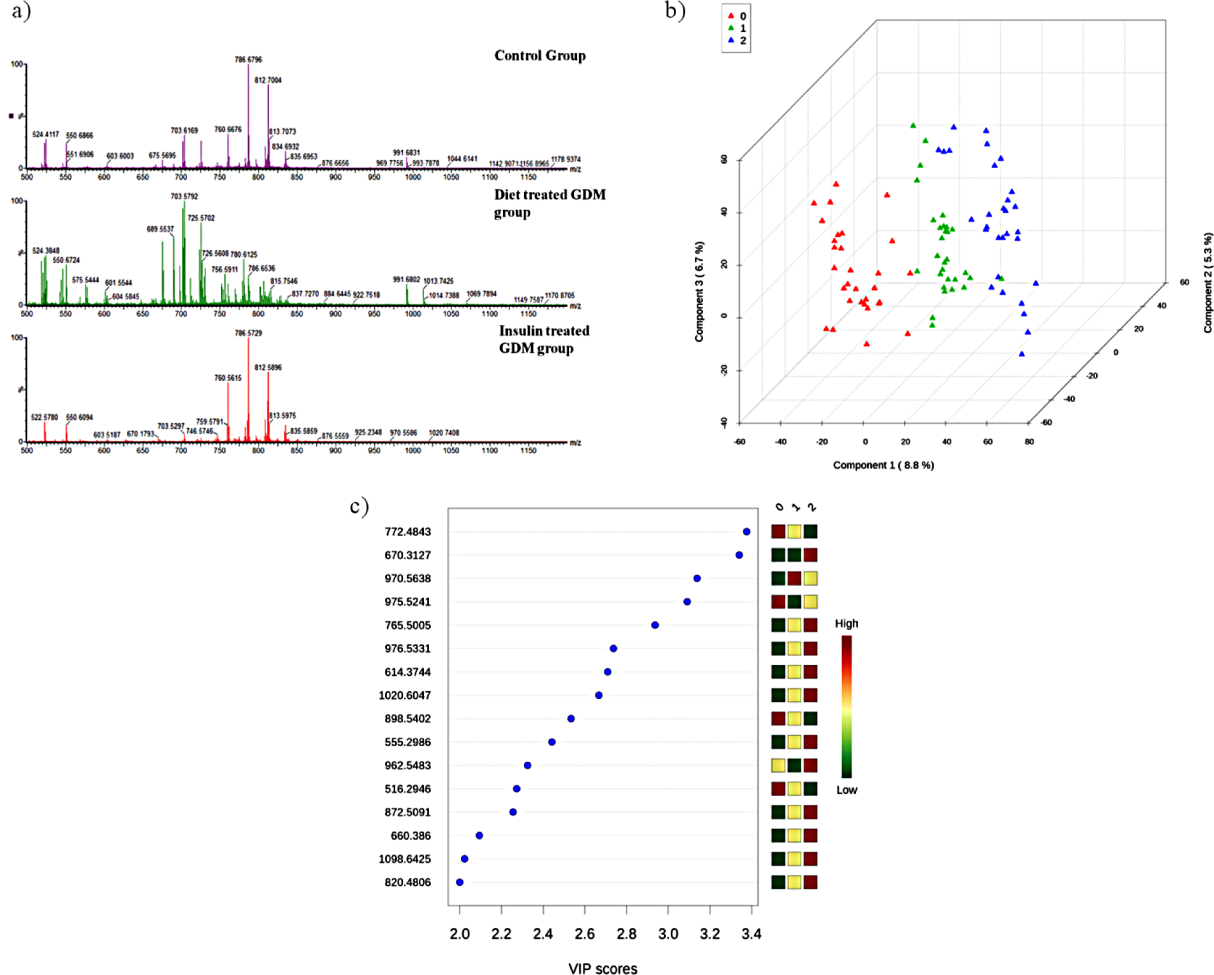
Serum lipid profile of healthy pregnant women and women with milder and more severe forms of GDM. **(**A) ESI-MS characteristic spectra of healthy controls, diet-treated GDM and insulin- treated groups. (B) 3D score plot between the selected principal components. (C) Important features identified by PLS-DA. The colored boxes on the right indicate the relative concentrations of the corresponding metabolite in each group under study. Group 0 = Control; Group 1 = Diet-treated GDM; Group 2 = Insulin-treated GDM.

The representative spectra of each group are shown in Fig 1A. One-way ANOVA analysis revealed 143 statistical significant ions, of which 93 were identified (Table 2). Of these, 4 were Fatty Acyls (FA), 1 Glycerolipid (GL), 63 Glycerophopholipids (GP), 5 Prenol Lipids (PR), 5 Sphingolipids (SL) and 15 Sterol Lipids (ST). Tukey’s HSD post-hoc test detected differences among the groups, but it was not possible to identify in which group the representation of each ion was higher or lower. A total of 14 ions (1 FA, 1 GL, 11 GP and 1 ST) characterized the diet-treated GDM group (diet-treated GDM x control). A total of 18 ions (10 GP, 2 PR, 2 SP and 4 SL) were differentially represented in the AIT-GDM group. There were 7 ions (5 GP and 2 ST) differentially represented in the two forms of GDM (diet-treated GDM versus AIT-GDM).

**Table 2:** Identified ions revealed by One-way ANOVA, sorting by Tukey’s HSD post-hoc test.

Mass (*m/z*)	Category	Formula	p value	Tukey's HSD
**599.5127**	Fatty Acyls	C_38_H_72_O_2_	0.023161	1–0
**603.5169**	Glycerolipids	C_37_H_72_O_4_	0.048001	1–0
**533.2781**	Glycerophospholipids	C_24_H_47_O_9_P	0.028454	1–0
**662.3447**	Glycerophospholipids	C_30_H_58_NO_10_P	0.0024812	1–0
**689.5498**	Glycerophospholipids	C_39_H_77_O_7_P	0.031083	1–0
**718.5735**	Glycerophospholipids	C_40_H_80_NO_7_P	0.042917	1–0
**721.3664**	Glycerophospholipids	C_33_H_63_O_13_P	0.044215	1–0
**749.5307**	Glycerophospholipids	C_40_H_77_O_10_P	0.015158	1–0
**756.6054**	Glycerophospholipids	C_43_H_82_NO_7_P	0.023612	1–0
**794.6333**	Glycerophospholipids	C_46_H_84_NO_7_P	0.035427	1–0
**850.6029**	Glycerophospholipids	C_48_H_84_NO_9_P	0.019524	1–0
**981.4979**	Glycerophospholipids	C_53_H_83_O_13_P	0.036555	1–0
**1003.5222**	Glycerophospholipids	C_53_H_89_O_13_P	0.031282	1–0
**713.3889**	Sterol Lipids	C_36_H_56_O_14_	0.033264	1–0
**506.2777**	Glycerophospholipids	C_22_H_46_NO_8_P	0.0017259	2–0
**544.3917**	Glycerophospholipids	C_27_H_56_NO_6_P	0.0094764	2–0
**586.3184**	Glycerophospholipids	C_28_H_54_NO_7_P	0.0018382	2–0
**606.3403**	Glycerophospholipids	C_27_H_54_NO_9_P	0.023815	2–0
**632.4543**	Glycerophospholipids	C_33_H_62_NO_8_P	0.0043383	2–0
**660.386**	Glycerophospholipids	C_31_H_60_NO_10_P	0.00017847	2–0
**675.53**	Glycerophospholipids	C_38_H_75_O_7_P	0.00064645	2–0
**812.6175**	Glycerophospholipids	C_46_H_86_NO_8_P	0.04182	2–0
**844.5894**	Glycerophospholipids	C_49_H_82_NO_8_P	0.003992	2–0
**884.5675**	Glycerophospholipids	C_50_H_78_NO_10_P	0.02529	2–0
**531.3707**	Prenol Lipids	C_32_H_50_O_6_	0.0038973	2–0
**943.478**	Prenol Lipids	C_52_H_72_O_14_	0.02148	2–0
**824.5521**	Sphingolipids	C_42_H_81_NO_12_S	0.028795	2–0
**991.6659**	Sphingolipids	C_51_H_94_N_2_O_16_	0.0063787	2–0
**507.3635**	Sterol Lipids	C_31_H_48_O_4_	0.0094738	2–0
**558.3406**	Sterol Lipids	C_29_H_51_NO_7_S	0.0073947	2–0
**933.4854**	Sterol Lipids	C_46_H_76_O_19_	0.0040151	2–0
**1047.6096**	Sterol Lipids	C_52_H_86_O_21_	0.0015661	2–0
**585.3006**	Glycerophospholipids	C_26_H_49_O_12_P	0.0036497	2–1
**619.3145**	Glycerophospholipids	C_29_H_47_O_12_P	0.015053	2–1
**661.4505**	Glycerophospholipids	C_36_H_69_O_8_P	0.034327	2–1
**842.4538**	Glycerophospholipids	C_45_H_74_NO_10_P	0.027476	2–1
**961.5004**	Glycerophospholipids	C_50_H_83_O_13_P	0.018855	2–1
**595.3661**	Sterol Lipids	C_34_H_52_O_7_	0.0014459	2–1
**763.3888**	Sterol Lipids	C_39_H_64_O_13_	0.037829	2–1
**527.2826**	Fatty Acyls	C_23_H_42_O_13_	2.24E-05	1–0; 2–0
**518.3728**	Glycerophospholipids	C_25_H_54_NO_6_P	0.0012904	1–0; 2–0
**528.2808**	Glycerophospholipids	C_25_H_48_NO_7_P	0.011041	1–0; 2–0
**532.2779**	Glycerophospholipids	C_23_H_44_NO_9_P	0.0015486	1–0; 2–0
**685.474**	Glycerophospholipids	C_36_H_71_O_8_P	0.00066563	1–0; 2–0
**701.5422**	Glycerophospholipids	C_40_H_77_O_7_P	2.70E-05	1–0; 2–0
**703.5581**	Glycerophospholipids	C_40_H_79_O_7_P	0.0022506	1–0; 2–0
**708.5282**	Glycerophospholipids	C_37_H_74_NO_9_P	0.0047033	1–0; 2–0
**720.4177**	Glycerophospholipids	C_35_H_69_O_8_PS	0.021746	1–0; 2–0
**746.4401**	Glycerophospholipids	C_40_H_70_NO_8_P	0.0081335	1–0; 2–0
**758.6159**	Glycerophospholipids	C_43_H_84_NO_7_P	0.00053932	1–0; 2–0
**765.5005**	Glycerophospholipids	C_39_H_73_O_12_P	3.41E-14	1–0; 2–0
**772.4843**	Glycerophospholipids	C_42_H_72_NO_8_P	2.96E-08	1–0; 2–0
**806.6279**	Glycerophospholipids	C_44_H_88_NO_9_P	7.35E-05	1–0; 2–0
**828.6145**	Glycerophospholipids	C_46_H_86_NO_9_P	0.00010221	1–0; 2–0
**900.5578**	Glycerophospholipids	C_52_H_80_NO_8_P	1.52E-05	1–0; 2–0
**975.5241**	Glycerophospholipids	C_46_H_82_O_16_P_2_	1.97E-19	1–0; 2–0
**976.5331**	Glycerophospholipids	C_44_H_81_N_3_O_15_P_2_	1.40E-12	1–0; 2–0
**601.5148**	Prenol Lipids	C_42_H_64_O_2_	0.00023122	1–0; 2–0
**944.5478**	Sphingolipids	C_48_H_91_NO_12_S	0.0010416	1–0; 2–0
**1112.6483**	Sphingolipids	C_55_H_101_NO_21_	0.00011821	1–0; 2–0
**555.2986**	Sterol Lipids	C_27_H_48_O_8_S	1.77E-06	1–0; 2–0
**573.3884**	Sterol Lipids	C_34_H_52_O_7_	0.0036142	1–0; 2–0
**588.3636**	Glycerophospholipids	C_28_H_56_NO_8_P	1.13E-07	1–0; 2–0; 2–1
**614.3744**	Glycerophospholipids	C_30_H_58_NO_8_P	2.81E-08	1–0; 2–0; 2–1
**898.5402**	Glycerophospholipids	C_50_H_86_NO_8_P	6.10E-08	1–0; 2–0; 2–1
**575.5295**	Fatty Acyls	C_36_H_72_O_3_	0.0056344	1–0; 2–1
**608.4196**	Glycerophospholipids	C_31_H_62_NO_8_P	0.0035127	1–0; 2–1
**639.3997**	Glycerophospholipids	C_33_H_61_O_8_P	0.00010065	1–0; 2–1
**694.4318**	Glycerophospholipids	C_36_H_66_NO_8_P	0.00036177	1–0; 2–1
**744.6102**	Glycerophospholipids	C_42_H_82_NO_7_P	8.67E-05	1–0; 2–1
**653.3326**	Prenol Lipids	C_39_H_50_O_7_	0.0065297	1–0; 2–1
**529.312**	Sterol Lipids	C_29_H_46_O_7_	7.95E-05	1–0; 2–1
**515.2811**	Fatty Acyl	C_25_H_42_N_2_O_7_S	0.001328	2–0; 2–1
**530.3062**	Glycerophospholipids	C_25_H_50_NO_7_P	0.00032057	2–0; 2–1
**568.3717**	Glycerophospholipids	C_27_H_54_NO_9_P	2.06E-06	2–0; 2–1
**578.2951**	Glycerophospholipids	C_25_H_50_NO_9_P	0.0012762	2–0; 2–1
**672.4383**	Glycerophospholipids	C_34_H_68_NO_8_P	0.00032939	2–0; 2–1
**739.4805**	Glycerophospholipids	C_37_H_71_O_12_P	0.00036796	2–0; 2–1
**820.4806**	Glycerophospholipids	C_43_H_76_NO_10_P	0.0011045	2–0; 2–1
**872.5091**	Glycerophospholipids	C_47_H_80_NO_10_P	7.66E-05	2–0; 2–1
**896.4743**	Glycerophospholipids	C_48_H_76_NO_10_P	0.002795	2–0; 2–1
**899.4582**	Glycerophospholipids	C_47_H_73_O_13_P	0.000357	2–0; 2–1
**1006.5383**	Glycerophospholipids	C_48_H_85_N_3_O_15_P_2_	0.00010404	2–0; 2–1
**1028.5152**	Glycerophospholipids	C_48_H_85_N_3_O_15_P_2_	0.014492	2–0; 2–1
**508.449**	Sphingolipids	C_32_H_61_NO_3_	0.0088719	2–0; 2–1
**514.2695**	Sterol Lipids	C_26_H_42_NO_7_S	1.02E-06	2–0; 2–1
**516.2946**	Sterol Lipids	C_26_H_45_NO_7_S	9.04E-07	2–0; 2–1
**541.2933**	Sterol Lipids	C_27_H_38_F_6_O_4_	0.00017966	2–0; 2–1
**741.4259**	Sterol Lipids	C_39_H_64_O_13_	0.0030906	2–0; 2–1
**819.4219**	Sterol Lipids	C_41_H_64_O_15_	0.0070071	2–0; 2–1
**580.302**	Glycerophospholipids	C_28_H_48_NO_8_P	0.037836	
**673.4076**	Glycerophospholipids	C_33_H_63_O_10_P	0.047301	
**623.5099**	Prenol Lipids	C_42_H_64_O_2_	0.040101	

Group 0 = Control; Group 1 = Diet-treated GDM; Group 2 = Insulin-treated GDM.

Twenty-three ions (1 FA, 17 GP, 1 PR, 2 SP and 2 ST) were characteristic of GDM, independent of treatment mode (diet-treated GDM x control and AIT-GDM x control). There were 7 ions (1 FA, 4 GP, 1 PR and 1 ST) specific to the diet-treated GDM group (diet-treated GDM x control and diet-treated GDM x AIT-GDM). On the other hand, 18 ions (1 FA, 11 GP, 1 SP and 5 ST) were differentially represented in the AIT-GDM group (AIT-GDM x control and AIT-GDM x diet-treated GDM). Finally, three ions classified as GP were differentially represented between the three groups. The ANOVA test detected a significant difference in three ions (2 GP and 1 PR) that was not confirmed by the post-hoc test (Table 2).

PCA analysis identified that the five first principal components (PC’s) explained 90.4% of the data model variance. Separation between the three groups was achieved by PLS-DA (Fig 1B). The sixteen most important ions for the discrimination of the groups were selected using the first 3 components (Fig 1C).

Only 11 ions of identified VIPs were classified: 9 GP and 2 ST (Table 3).

**Table 3:** Identified VIPs ions.

Mass (*m/z*)	Category	Formula
**772.4843**	Glycerophospholipids	C_42_H_72_NO_8_P
**670.3127**	Not identified	
**970.5638**	Not identified	
**975.5241**	Glycerophospholipids	C_46_H_82_O_16_P_2_
**765.5005**	Glycerophospholipids	C_39_H_73_O_12_P
**976.5331**	Glycerophospholipids	C_44_H_81_N_3_O_15_P_2_
**614.3744**	Glycerophospholipids	C_30_H_58_NO_8_P
**1020.6047**	Not identified	
**898.5402**	Glycerophospholipids	C_50_H_86_NO_8_P
**555.2986**	Sterol Lipids	C_27_H_48_O_8_S
**962.5483**	Not identified	
**516.2946**	Sterol Lipids	C_26_H_45_NO_7_S
**872.5091**	Glycerophospholipids	C_47_H_80_NO_10_P
**660.386**	Glycerophospholipids	C_31_H_60_NO_10_P
**1098.6425**	Not identified	
**820.4806**	Glycerophospholipids	C_43_H_76_NO_10_P

## Discussion

We detected clear differences in the serum lipid profile of healthy pregnant women compared to GDM patients and between milder (diet-treated) compared to more severe (AIT) forms of GDM.

The physiological dyslipidemia that occurs in normal pregnancy is apparently more intense in women with GDM, despite some controversies. Increased hyperlipidemia could be caused by a combination of IR associated with hormonal changes, such as decreased progesterone, prolactin and estradiol [9, 13].

To the best of our knowledge, this is the first study to evaluate the lipid fingerprint of GDM patients. A few studies have used this tool to assess the mechanisms involved in normal pregnancy and its complications. Durn et al. (2010) analyzed the myometrial prostanoid profile of women in labor and not in labor at term [24]. Other investigators reported significant differences in the lipid profile of syncytiotrophoblasts from placentas of women with preeclampsia and with recurrent abortion compared with healthy controls [25]. de Oliveira et al. (2012) identified lipid compounds in blood plasma from women with preeclampsia that could be associated with the disease [26]. This approach has also been used to investigate the role of lipids in Diabetes *Mellitus*. Recent studies with T2DM suggest that this approach is useful to characterize lipid changes in peripheral blood associated with the disease [16, 17].

Kaur et al. (2013) evaluated both the lipid and metabolic profiles of 69 patients with T2DM compared with 41 healthy individuals and reported hyper and hypo-represented lipids in the plasma of the diabetics, mostly glycerophospholipids (phosphatidylcholine, phosphatidylglycerol and phosphatidylethanolamine) and sphingolipids. The authors also reported that T2DM affects molecules involved in carbohydrate, amino acids and lipid metabolism [16].

Lipid profiling seems to bring more relevant information than gene expression analysis per se. Zhao et al. (2013) analyzed lipid profiles and gene expression in 84 T2DM and 60 healthy controls. While gene expression evaluations alone revealed no significant differences between patients, lipid profile analyses detected different characteristic features in each group. Lipid profiling can also contribute to the interpretation of gene expression results allowing the identification of biological pathways and suspected genes involved in insulin resistance [17].

Merzouk et al. (2000) reported altered lipid profiles in poorly controlled pregnant T1DM patients [27]. Changes in lipids in early pregnancy may be related with higher risks of IR and hyperglycemia later in gestation. On the other hand, GDM patients that require insulin therapy to achieve glycemic control have more severe clinical manifestations of the disease and higher risks for metabolic disorders after pregnancy [5, 6, 28].

Based on the lipid extraction protocol and ESI-(+)-MS method used in this study, we expected to find a considerable number of ions classified as Glycerophospholipids. Saccharolipids, Polyketides and Carnitine classes in SimLipid search were not included due the characteristics of the protocol. It was possible to identify ions in several lipid classes. Our approach did not allow the identification of the specific lipid that corresponds to each ion, but we were able to identify the lipid classes.

Most of the ions (63) belonged to the GP class. The most common GP in mammalian cell membrane are glycerophosphatidic acids, glycerophosphocholines, glycerophosphoethanolamines, glycerophosphoinositols, glycerophosphoglycerols, glycerophosphoserines, and cardiolipins [29]. GPs, key components of the cell membrane, are involved in several mechanisms including anti-inflammatory and immunomodulatory activities [30]. GPs also seem to have beneficial effects in the treatment of dyslipidemias, but it is still unclear which specific mechanisms and subclasses are involved in this process [31, 32].

Sterol Lipids were the second class most frequently observed in this study (15 ions). They are important constituents of the cell membrane, involved in cell growth and proliferation and are precursors of bile acids and steroid hormones [33]. Cholesterol is considered the major element of this class.

We also identified five ions in the Sphingolipids and Prenol Lipids. Sphingolipids are involved in the cascade of intracellular signaling and in cell recognition. Derivatives of glycosylated sphingolipid, called glycosphingolipids, are the class of more complex and structurally diverse SP, but there are other subclasses, such as sphingoid bases, ceramides and others [29, 34]. Some lipids of this class have currently been associated with glucose metabolism and insulin resistance [35, 36]. Prenol Lipids are vital for cell survival and are precursors of many vitamins, such as vitamin A and E [29].

Our analysis also detected Fatty Acyl (4 ions) and Glycerolipid (1 íon). Fatty Acyls are responsible for body energy and the formation of complex lipids [29]. This class of lipids is influenced by diet and insulin can block the release of these lipids. Moreover, obesity seems to increase free FA. Finally, Glycerolipids are involved in a large number of biochemical functions, from energy reserve to serving as precursors of intracellular signaling after activation of membrane receptors. This class of lipids is divided in subgroups, such as triacylglycerols (TAG) and diacylglycerols (DAG) [37]. TAGs are the major source of cellular energy, serving as a source of essential and non-essential fatty acids, as well as serving as precursors in the biosynthesis of phospholipids [38]. On the other hand, DAGs are intracellular messengers that have been identified as mediators of IR [39].

Due to technical limitations, it was not possible to classify all statistically significant ions. However, we were able to categorize most of the ions identified into a lipid classification. Nevertheless, the next step should be identify and quantify the specific ions that were differently presented in each group. We intend to perform MS/MS analysis of all VIP ions. After these tests we should be able to identify the most important ions to screen in order to characterize and understand the role of lipids in the physiopathology of GDM. This specific analysis is challenging because it requires a larger volume of serum, additional testing using other technical approaches and is very expensive.

Another point to be considered is that we did not collect fasting blood samples. This, in theory, could have affected our results. However, all samples were collected in the afternoon, within the first 2 hours after lunch thus reducing the possible heterogeneity related to this aspect. Moreover, all the patients with GDM were following exactly the same type of diet and therefore the caloric intake and composition of that meal was probably very similar.

Despite these limitations, this study was the first to use the lipid fingerprinting approach in women with GDM, supporting the relevant role of lipid profile in the pathogenesis of this disease.

There are marked differences in lipid fingerprinting between healthy pregnant women compared to those with GDM in the third trimester. Moreover, the lipid profile of women with more severe forms of GDM differs considerably from that of women with milder forms of GDM.

Historically, to optimize maternal and perinatal outcomes, obese and high risk pregnant women are counselled to follow healthy diets with controlled caloric intake and to exercise. However, the effects of these recommendations on metabolic (glycemic and lipid) parameters are still controversial. While some investigators reported significant changes in the lipid profile of women on low carbohydrate diets [40–42] and receiving insulin therapy [43, 44], other studies, including a systematic review, did not find a significant effect of these interventions on lipid metabolism [19, 45–47]. Thus, although it is possible that part of the differences in the lipid profiles of our groups could be due to the treatment itself, this hypothesis is not currently supported by evidence.

This study identified some lipids that should be further investigated to clarify the pathogenesis of GDM. Our results indicate that a lipid fingerprinting approach could be further developed and tested in the future as a potential new tool to help predict the risk of developing more severe forms of GDM.

## References

[1] American Diabetes Association. Diagnosis and classification of diabetes mellitus. Diabetes Care. 2011;34 Suppl 1:S62–69. 10.2337/dc11-S062 21193628 PMC3006051

[2] International Association of Diabetes and Pregnancy Study Groups Consensus Panel, Metzger BE, Gabbe SG, Persson B, Buchanan TA, Catalano PA, et al International association of diabetes and pregnancy study groups recommendations on the diagnosis and classification of hyperglycemia in pregnancy. Diabetes Care. 2010;33(3):676–682. 10.2337/dc09-1848 20190296 PMC2827530

[3] Coustan DR. Gestational diabetes mellitus. Clin Chem. 2013;59(9):1310–1321. 10.1373/clinchem.2013.203331 23536513

[4] Landon MB, Spong CY, Thom E, Carpenter MW, Ramin SM, Casey B, et al A multicenter, randomized trial of treatment for mild gestational diabetes. N Engl J Med. 2009;361(14):1339–1348. 10.1056/NEJMoa0902430 19797280 PMC2804874

[5] Bayraktar F, Akinci B, Celtik A, Tunali S, Genc S, Ozcan MA, et al Insulin need in gestational diabetes is associated with a worse cardiovascular risk profile after pregnancy. Intern Med. 2012;51(8):839–843. 22504236 10.2169/internalmedicine.51.5846

[6] Sokup A, Ruszkowska-Ciastek B, Goralczyk K, Walentowicz MG, Szyma Ski M, Ro D. Insulin resistance as estimated by the homeostatic method at diagnosis of gestational diabetes: estimation of disease severity and therapeutic needs in a population-based study. BMC Endocr Disord. 2013;13(1):21. 23819910 10.1186/1472-6823-13-21 PMC3702418

[7] Ben-Haroush A, Yogev Y, Hod M. Epidemiology of gestational diabetes mellitus and its association with Type 2 diabetes. Diabet Med. 2004;21(2):103–113. 14984444 10.1046/j.1464-5491.2003.00985.x

[8] Saisho Y, Miyakoshi K, Tanaka M, Shimada A, Ikenoue S, Kadohira I, et al Beta cell dysfunction and its clinical significance in gestational diabetes. Endocr J. 2010;57(11):973–980. 20847480 10.1507/endocrj.k10e-231

[9] Herrera E, Ortega-Senovilla H. Disturbances in lipid metabolism in diabetic pregnancy—Are these the cause of the problem? Best Pract Res Clin Endocrinol Metab. 2010;24(4):515–525. 10.1016/j.beem.2010.05.006 20832733

[10] Murakami M. Lipid mediators in life science. Exp Anim. 2011;60(1):7–20. 21325748 10.1538/expanim.60.7

[11] Riemens SC, van Tol A, Scheek LM, Dullaart RP. Plasma cholesteryl ester transfer and hepatic lipase activity are related to high-density lipoprotein cholesterol in association with insulin resistance in type 2 diabetic and non-diabetic subjects. Scand J Clin Lab Invest. 2001;61(1):1–9. 11300605 10.1080/00365510151067866

[12] Borggreve SE, De Vries R, Dullaart RP. Alterations in high-density lipoprotein metabolism and reverse cholesterol transport in insulin resistance and type 2 diabetes mellitus: role of lipolytic enzymes, lecithin:cholesterol acyltransferase and lipid transfer proteins. Eur J Clin Invest. 2003;33(12):1051–1069. 14636288 10.1111/j.1365-2362.2003.01263.x

[13] Montelongo A, Lasuncion MA, Pallardo LF, Herrera E. Longitudinal study of plasma lipoproteins and hormones during pregnancy in normal and diabetic women. Diabetes. 1992;41(12):1651–1659. 1446807 10.2337/diab.41.12.1651

[14] Ikeda K, Oike Y, Shimizu T, Taguchi R. Global analysis of triacylglycerols including oxidized molecular species by reverse-phase high resolution LC/ESI-QTOF MS/MS. J Chromatogr B Analyt Technol Biomed Life Sci. 2009;877(25):2639–2647. 10.1016/j.jchromb.2009.03.047 19481987

[15] Han X, Gross RW. Global analyses of cellular lipidomes directly from crude extracts of biological samples by ESI mass spectrometry: a bridge to lipidomics. J Lipid Res. 2003;44(6):1071–1079. 12671038 10.1194/jlr.R300004-JLR200

[16] Kaur P, Rizk N, Ibrahim S, Luo Y, Younes N, Perry B, et al Quantitative metabolomic and lipidomic profiling reveals aberrant amino acid metabolism in type 2 diabetes. Mol Biosyst. 2013;9(2):307–317. 10.1039/c2mb25384d 23247761

[17] Zhao C, Mao J, Ai J, Shenwu M, Shi T, Zhang D, et al Integrated lipidomics and transcriptomic analysis of peripheral blood reveals significantly enriched pathways in type 2 diabetes mellitus. BMC Med Genomics. 2013;6 Suppl 1:S12 10.1186/1755-8794-6-S1-S12 23369247 PMC3552685

[18] Giuffrida FM, Guedes AD, Rocco ER, Mory DB, Dualib P, Matos OS, et al Heterogeneous behavior of lipids according to HbA1c levels undermines the plausibility of metabolic syndrome in type 1 diabetes: data from a nationwide multicenter survey. Cardiovasc Diabetol. 2012;11:156 10.1186/1475-2840-11-156 23270560 PMC3547761

[19] Karkkainen H, Laitinen T, Heiskanen N, Saarelainen H, Valtonen P, Lyyra-Laitinen T, et al Need for insulin to control gestational diabetes is reflected in the ambulatory arterial stiffness index. BMC Pregnancy Childbirth. 2013;13:9 10.1186/1471-2393-13-9 23324111 PMC3556301

[20] Mendez-Figueroa H, Daley J, Lopes VV, Coustan DR. Predicting the need for medical therapy in patients with mild gestational diabetes. Am J Perinatol. 2014;31(2):105–112. 10.1055/s-0033-1338174 23508701

[21] Bakiner O, Bozkirli E, Ozsahin K, Sariturk C, Ertorer E. Risk Factors That can Predict Antenatal Insulin Need in Gestational Diabetes. J Clin Med Res. 2013;5(5):381–388. 10.4021/jocmr1515w 23976911 PMC3748663

[22] Pertot T, Molyneaux L, Tan K, Ross GP, Yue DK, Wong J. Can common clinical parameters be used to identify patients who will need insulin treatment in gestational diabetes mellitus? Diabetes Care. 2011;34(10):2214–2216. 10.2337/dc11-0499 21836104 PMC3177752

[23] Bligh EG, Dyer WJ. A rapid method of total lipid extraction and purification. Can J Biochem Physiol. 1959;37(8):911–917. 13671378 10.1139/o59-099

[24] Durn JH, Marshall KM, Farrar D, O'Donovan P, Scally AJ, Woodward DF, et al Lipidomic analysis reveals prostanoid profiles in human term pregnant myometrium. Prostaglandins Leukot Essent Fatty Acids. 2010;82(1):21–26. 10.1016/j.plefa.2009.11.002 19954938

[25] Baig S, Lim JY, Fernandis AZ, Wenk MR, Kale A, Su LL, et al Lipidomic analysis of human placental syncytiotrophoblast microvesicles in adverse pregnancy outcomes. Placenta. 2013;34(5):436–442. 10.1016/j.placenta.2013.02.004 23465879

[26] De Oliveira L, Camara NO, Bonetti T, Lo Turco EG, Bertolla RP, Moron AF, et al Lipid fingerprinting in women with early-onset preeclampsia: a first look. Clin Biochem. 2012;45(10–11):852–855. 10.1016/j.clinbiochem.2012.04.012 22548912

[27] Merzouk H, Madani S, Korso N, Bouchenak M, Prost J, Belleville J. Maternal and fetal serum lipid and lipoprotein concentrations and compositions in type 1 diabetic pregnancy: relationship with maternal glycemic control. J Lab Clin Med. 2000;136(6):441–448. 11128745 10.1067/mlc.2000.111004

[28] Lee AJ, Hiscock RJ, Wein P, Walker SP, Permezel M. Gestational diabetes mellitus: clinical predictors and long-term risk of developing type 2 diabetes: a retrospective cohort study using survival analysis. Diabetes Care. 2007;30(4):878–883. 17392549 10.2337/dc06-1816

[29] Bou Khalil M, Hou W, Zhou H, Elisma F, Swayne LA, Blanchard AP, et al Lipidomics era: accomplishments and challenges. Mass Spectrom Rev. 2010;29(6):877–929. 10.1002/mas.20294 20931646

[30] Sahebkar A. Fat lowers fat: purified phospholipids as emerging therapies for dyslipidemia. Biochim Biophys Acta. 2013;1831(4):887–893. 10.1016/j.bbalip.2013.01.013 23354177

[31] Cohn JS, Wat E, Kamili A, Tandy S. Dietary phospholipids, hepatic lipid metabolism and cardiovascular disease. Curr Opin Lipidol. 2008;19(3):257–262. 10.1097/MOL.0b013e3282ffaf96 18460916

[32] Kullenberg D, Taylor LA, Schneider M, Massing U. Health effects of dietary phospholipids. Lipids Health Dis. 2012;11:3 10.1186/1476-511X-11-3 22221489 PMC3316137

[33] Bhatti HN, Khera RA. Biological transformations of steroidal compounds: a review. Steroids. 2012;77(12):1267–1290. 10.1016/j.steroids.2012.07.018 22910289

[34] Lahiri S, Futerman AH. The metabolism and function of sphingolipids and glycosphingolipids. Cell Mol Life Sci. 2007;64(17):2270–2284. 17558466 10.1007/s00018-007-7076-0 PMC11136246

[35] Hla T, Dannenberg AJ. Sphingolipid signaling in metabolic disorders. Cell Metab. 2012;16(4):420–434. 10.1016/j.cmet.2012.06.017 22982021 PMC3466368

[36] Summers SA. Sphingolipids and insulin resistance: the five Ws. Curr Opin Lipidol. 2010;21(2):128–135. 10.1097/MOL.0b013e3283373b66 20216312

[37] Murphy RC, Leiker TJ, Barkley RM. Glycerolipid and cholesterol ester analyses in biological samples by mass spectrometry. Biochim Biophys Acta. 2011;1811(11):776–783. 10.1016/j.bbalip.2011.06.019 21757029 PMC3205286

[38] Coleman RA, Lee DP. Enzymes of triacylglycerol synthesis and their regulation. Prog Lipid Res. 2004;43(2):134–176. 14654091 10.1016/s0163-7827(03)00051-1

[39] Coen PM, Goodpaster BH. Role of intramyocelluar lipids in human health. Trends Endocrinol Metab. 2012;23(8):391–398. 10.1016/j.tem.2012.05.009 22721584 PMC4908975

[40] Sharman MJ, Gomez AL, Kraemer WJ, Volek JS. Very low-carbohydrate and low-fat diets affect fasting lipids and postprandial lipemia differently in overweight men. J Nutr. 2004;134(4):880–885. 15051841 10.1093/jn/134.4.880

[41] Volek JS, Sharman MJ, Gomez AL, DiPasquale C, Roti M, Pumerantz A, et al Comparison of a very low-carbohydrate and low-fat diet on fasting lipids, LDL subclasses, insulin resistance, and postprandial lipemic responses in overweight women. J Am Coll Nutr. 2004;23(2):177–184. 15047685 10.1080/07315724.2004.10719359

[42] Schroeder N, Park YH, Kang MS, Kim Y, Ha GK, Kim HR, et al A randomized trial on the effects of 2010 Dietary Guidelines for Americans and Korean diet patterns on cardiovascular risk factors in overweight and obese adults. J Acad Nutr Diet. 2015;115(7):1083–1092. 10.1016/j.jand.2015.03.023 26115560

[43] Ponssen HH, Elte JW, Lehert P, Schouten JP, Bets D. Combined metformin and insulin therapy for patients with type 2 diabetes mellitus. Clin Ther. 2000;22(6):709–718. 10929918 10.1016/S0149-2918(00)90005-5

[44] Lau AN, Tang T, Halapy H, Thorpe K, Yu CH. Initiating insulin in patients with type 2 diabetes. CMAJ. 2012;184(7):767–776. 10.1503/cmaj.110779 22470171 PMC3328521

[45] Markovic TP, Muirhead R, Overs S, Ross GP, Louie JC, Kizirian N, et al Randomized Controlled Trial Investigating the Effects of a Low-Glycemic Index Diet on Pregnancy Outcomes in Women at High Risk of Gestational Diabetes Mellitus: The GI Baby 3 Study. Diabetes Care. 2015; 10.2337/dc15-0572 26185283

[46] Bravata DM, Sanders L, Huang J, Krumholz HM, Olkin I, Gardner CD, et al Efficacy and safety of low-carbohydrate diets: a systematic review. JAMA. 2003;289(14):1837–1850. 12684364 10.1001/jama.289.14.1837

[47] Vinter CA, Jorgensen JS, Ovesen P, Beck-Nielsen H, Skytthe A, Jensen DM. Metabolic effects of lifestyle intervention in obese pregnant women. Results from the randomized controlled trial 'Lifestyle in Pregnancy' (LiP). Diabet Med. 2014;31(11):1323–1330. 10.1111/dme.12548 24989831

